# Complete Genome Sequence of a Bovine Viral Diarrhea Virus Subgenotype 1g Strain Isolated in Italy

**DOI:** 10.1128/genomeA.00263-17

**Published:** 2017-04-27

**Authors:** Moira Bazzucchi, Luigi Bertolotti, Monica Giammarioli, Elisabetta Rossi, Stefano Petrini, Sergio Rosati, Gian Mario De Mia

**Affiliations:** a Istituto Zooprofilattico Sperimentale dell’Umbria e delle Marche, Perugia, Italy; b Dipartimento di Scienze Veterinarie, Università degli Studi di Torino, Grugliasco, Torino, Italy; c Dipartimento di Medicina Veterinaria, Università degli Studi di Perugia, Perugia, Italy

## Abstract

We report here the full-length sequence of the bovine viral diarrhea virus (BVDV) strain UM/103/04, isolated from a persistently infected cow in Italy. It belongs to the subgenotype 1g, which is described as a sporadic subgenotype in Italy. This is the first report of a complete genome sequence of BVDV-1g.

## GENOME ANNOUNCEMENT

Bovine viral diarrhea virus (BVDV) belongs to the genus *Pestivirus*, family *Flaviviridae* (1). It is a single-stranded RNA virus with a genome length of about 12.5 kb, organized as an open reading frame flanked by 5′ and 3′ untranslated regions (UTR) (2). Based on antigenic and nucleotide differences, three species of BVDV have been recognized: BVDV-1, BVDV-2, and HoBi-like, tentatively named BVDV-3 (3, 4). BVDV-1 contains 21 subgenotypes (BVDV-1a to BVDV-1u) (4–9). In Italy, BVDV-1a, BVDV-1b, and BVDV-1e were the predominant subgenotypes; the others have been reported to be less prevalent in the cattle population (10, 11). In the current study, we determined the full-length genome of a BVDV-1g strain.

The UM/103/04 isolate was collected in 2004 from a persistently infected animal. The virus was isolated from blood on a Madin-Darby bovine kidney (MDBK) cell line. The viral RNA has been extracted with the QIAamp viral RNA minikit from cell supernatant after appropriate enzymatic digestion. The quality of the total RNA was verified using a 2200 TapeStation RNA screen tape device (Agilent, Santa Clara, CA, USA) and its concentration ascertained using an ND-1000 spectrophotometer (NanoDrop, Wilmington, DE). The cDNA libraries were constructed by PGP using the Illumina TruSeq RNA sample prep kit (Illumina, San Diego, CA, USA), according to the manufacturer’s protocol, with minor modifications. The resulting double-stranded (ds) cDNA was end-repaired and adenylated. The Illumina adapter was added as indicated in the TruSeq RNA protocol. The quality of the libraries was determined via the Agilent 2200 TapeStation using high-sensitivity D1000 screen tape and quantified by the ABI9700 quantitative PCR (qPCR) instrument using the Kapa library quantification kit in triplicate, according to the manufacturer’s protocol (Kapa Biosystems, Woburn, MA, USA). Five microliters of the pooled library at a final concentration of 2 nM was used for sequencing using Illumina MiSeq with a 150 paired-end-read sequencing module. The reads generated were quality checked and assembled into contigs by *de novo* assembly algorithm using the analytical tools ABySS, Velvet, and Mira, integrated into Geneious (version 9.1.2).

The complete genome of the strain UM/103/04 comprises 12,164 nucleotides (nt), with 5′ and 3′ untranslated regions (UTRs) of 318 nt and 149 nt, respectively. The single large open reading frame encodes 3,898 amino acids. Compared to other subgenotypes, the virus shares only 77.00% to 84.10% nucleotide similarity with other published full-length BVDV-1 genomes. The large open reading frame encodes four structural proteins (C [nt 823 to 1128], E^rns^ [nt 1129 to 1809], E1 [nt 1810 to 2394], and E2 [nt 2395 to 3516]) and seven nonstructural proteins (N^pro^ [nt 319 to 822], p7 [nt 3517 to 3726], NS2/3 [nt 3727 to 7134], NS4A [nt 7135 to 7326], NS4B [nt 7327 to 8367], NS5A [nt 8368 to 9855], and NS5B [nt 9856 to 12012]). This study is the first report of the whole-genome sequence of BVDV-1g. Therefore, its publication represents an important contribution to filling the gap on molecular knowledge of BVDV-1.

### Accession number(s).

The genomic sequence of strain UM/103/04 has been deposited in GenBank under the accession no. LT797813.
